# 3D‐printed custom ankle braces for people with Charcot‐Marie‐Tooth disease: A pilot study

**DOI:** 10.1002/jfa2.70013

**Published:** 2024-10-18

**Authors:** Adam Philps, Mike Frecklington, Sarah Stewart

**Affiliations:** ^1^ Masterton Foot Clinic Masterton New Zealand; ^2^ School of Clinical Sciences Faculty of Health and Environmental Sciences Auckland University of Technology Auckland New Zealand

**Keywords:** ankle brace, balance, Charcot‐Marie‐Tooth disease, foot pain

## Abstract

**Introduction:**

Charcot‐Marie‐Tooth disease (CMT) is a neurodegenerative condition resulting in footdrop, ankle instability and impaired balance and gait. This study aimed to determine (1) whether 3D‐printed custom ankle braces improve function and balance in people with CMT and (2) whether this is an acceptable device for use in this population.

**Methods:**

A within‐subject comparison pragmatic/pilot study was undertaken. Ten people with CMT (mean [SD] age 48 [14] years, 60% male) were fitted with 3D‐printed ankle braces. Following a 4‐week wear‐in period, walking and balance tests and patient‐reported outcomes were assessed in two experimental conditions: (i) usual shoes and (ii) usual shoes with 3D‐printed custom ankle braces. Differences in outcome measures between experimental conditions were analysed using linear mixed models. Comfort, aesthetics and overall satisfaction of the brace were assessed via 100‐mm visual analogue scale (VAS). Adverse events and tripping/falls associated with the brace during the wear‐in period were also recorded by participants using daily diaries.

**Results:**

A significant improvement was seen during single‐leg balance with eyes open (*p* = 0.026, Cohen's *d* = 0.55) and a significant reduction in foot pain (*p* = 0.045, Cohen's *d* = 0.82), with use of the ankle brace. Mean (SD) 100 mm VAS scores were 62.7 mm (17.9) for overall comfort and 73.9 mm (21.2) for overall satisfaction. Subjective data from the daily dairies showed that one participant found the brace too firm around the ankle due to loss of soft tissue mass and two participants found it challenging to don and doff the brace due to loss of hand dexterity.

**Conclusion:**

This pilot study suggests that a 3D‐printed custom ankle brace may improve balance and reduce foot pain in people with CMT; however, larger‐scale trials are needed to further explore the impact of this brace on function and balance. Further customisation of the brace may also be required to improve acceptability for some people.

## 1 INTRODUCTION

Charcot‐Marie‐Tooth disease (CMT) is the most common hereditary motor sensory neuropathy [1]. The clinical presentation of CMT is characterised by motor and sensory nerve manifestations, with distal limb muscle atrophy being the most common initial presentation [2]. Pes cavus and hammer toes are seen in 71% of people with CMT [3] due to a muscular imbalance between the tibialis anterior and peroneus longus muscles, as well as progressive atrophy of the intrinsic foot muscles [4, 5]. Consequently, people with CMT experience major functional disability. Laboratory‐based gait analyses have identified altered gait in people with CMT, specifically, a loss of sagittal plane ankle stability [6] and footdrop resulting in a lack of heel contact during contact and a loss of propulsive power [7]. These inefficient gait strategies contribute to increased incidence of tripping and falls, increased energy consumption during walking and low levels of daily activity [7, 8].

Ankle foot orthoses are commonly prescribed in people with CMT to improve gait efficiency and reduce the risk of tripping and falls associated with footdrop and instability [9, 10, 11]. Traditional ankle foot orthoses comprises thermoplastic materials supporting the lower leg, ankle and foot. Despite the potential benefits offered by these traditional orthoses, patient compliance and satisfaction are poor [12, 13, 14]. Both prefabricated and customised ankle foot orthoses (which are made from casting the patients foot) have been associated with reports of dissatisfaction due to discomfort, aesthetics and the requirement to wear them with custom‐made orthopaedic footwear [9, 12, 13, 14]. People with CMT have also reported pain and skin irritation with the use of ankle foot orthoses, as well as damage to clothes from the hard surfaces and bulkiness/geometry of the devices [14].

Modern technology using computer aided design and manufacturing techniques have shown promise in the fabrication of three dimensional (3D)‐printed ankle foot orthoses [15], which have the potential to provide a fully customised device that is lightweight and discreet. However, further research is needed to evaluate the use of 3D scanning and printing of ankle brace devices for people with CMT. The aims of this pilot study were (1) to determine preliminary effectiveness of a 3D‐printed custom ankle brace on lower limb function in people with CMT and (2) to determine patient‐perceived acceptability and satisfaction of the ankle brace for people with CMT.

## 2 MATERIALS AND METHODS

### 2.1 Study design

Participants served as their own controls in a within‐group comparison pragmatic/pilot study. Participants were tested in two experimental conditions: (i) in their usual footwear and (ii) in their usual footwear with 3D‐printed custom ankle braces.

### 2.2 Participants

Participants were recruited via the Muscular Dystrophy New Zealand CMT disease support group, Facebook CMT disease New Zealand support group and through professional links with local orthopaedic surgeons, neurologists and Podiatry New Zealand. Participants were included if they were over 16 years of age, had a self‐reported physician diagnosis of CMT, were able to walk independently or had unilateral or bilateral evidence of footdrop. Footdrop was assessed clinically by a single experienced podiatrist (AP) with >20 years of clinical experience. Footdrop was defined as a lack of heel contact during the contact phase of gait and subsequent loss of propulsive power. Participants also needed to be willing to travel to one of the three study locations for study visits. Eligibility was not affected by the use of a walking aid. Participants were excluded if they had received prescription of foot/ankle orthoses in the past 3 months. Ethical approval was obtained from AUT Ethics Committee (AUTEC 22/91). All participants were required to provide written informed consent prior to participation.

### 2.3 Data collection

All data were collected between November 2022 and March 2023 by a single experienced podiatrist (AP) (>20 years clinical experience) at one of the three locations across Aotearoa New Zealand: Masterton Foot Clinic (Masterton), Silverstream Podiatry Clinic (Wellington) and McRae Podiatry (Palmerston North). At the initial study visit, demographic and medical characteristics were recorded. In addition, foot type was assessed using the foot posture index [16], which provided an overall score for each foot ranging from −12 (highly supinated) to +12 (highly pronated). Plantar protective sensation was assessed using a 10 g Semmes–Weinstein monofilament at three sites on the plantar foot (hallux, first metatarsal head and fifth metatarsal head) [17]. Each site was assessed once and loss of sensation for each foot was defined as an inability to detect the monofilament at ≥1 sites.

### 2.4 Experimental conditions

#### 2.4.1 Usual footwear

Due to the pragmatic nature of this study that aimed to reflect routine clinical practice, there was no standardisation for footwear, and participants were instructed to wear their own choice of athletic style footwear during all study assessments.

#### 2.4.2 Usual footwear with 3D‐scanned and 3D‐printed custom‐made braces

**FIGURE 1:**
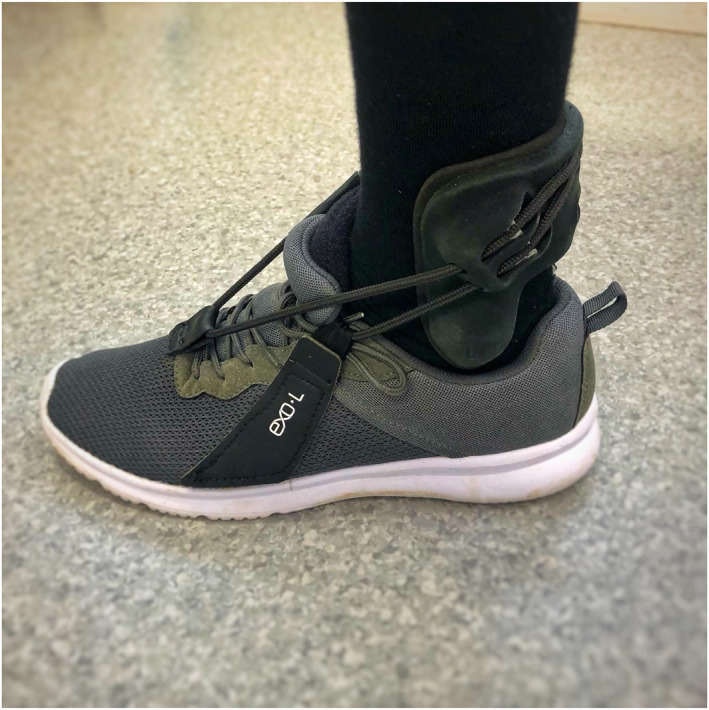
EXO‐L UP ankle brace.

**FIGURE 2:**
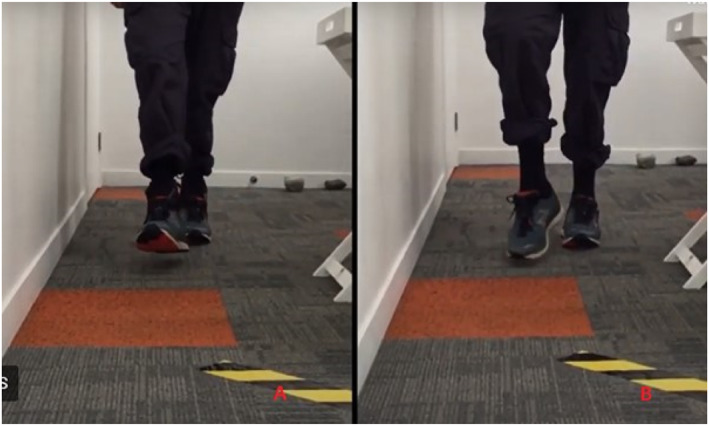
Use of the EXO‐L UP brace in a participant with Charcot‐Marie‐Tooth. (A) Ankle dorsiflexion and ground clearance with the EXO‐L UP brace; (B) Footdrop without EXO‐L UP brace (Images shared with permission).

**FIGURE 3:**
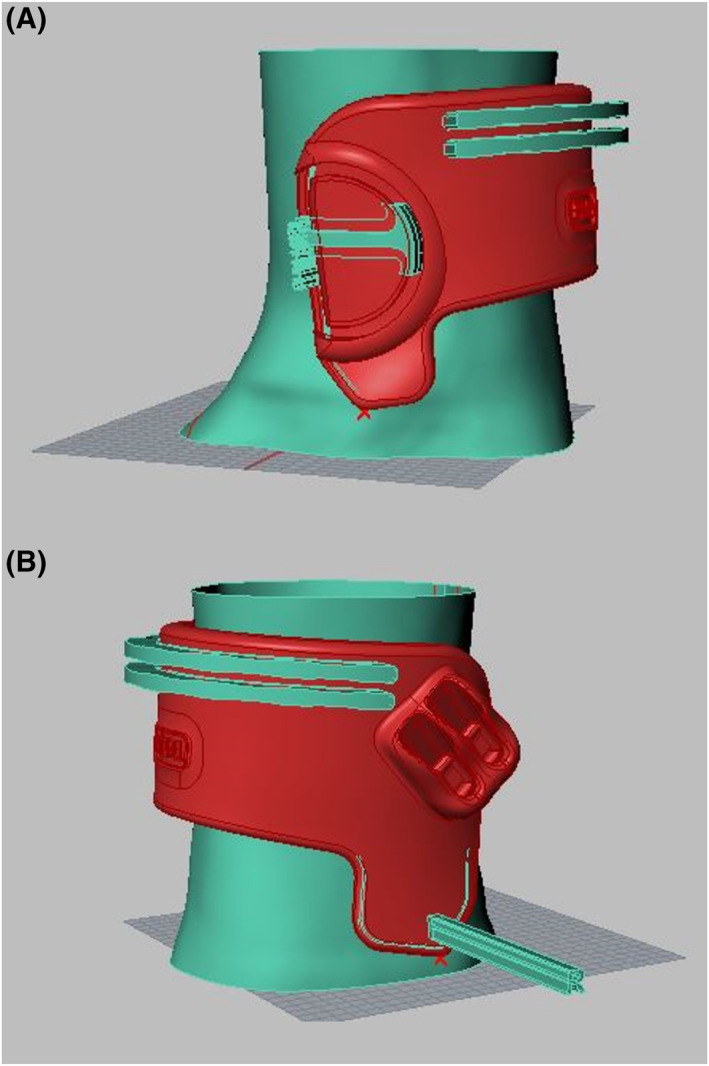
Digital design images of 3D EXO‐L UP brace showing medial (A) and lateral (B) views.

All participants received 3D‐scanned and 3D‐printed custom‐made braces (EXO‐L UP, Brace for Innovation) (Figure 1). This brace, which has recently been developed, provides an individualised fit to the foot and shoe. The brace consists of a thermoplastic nylon (PA11) shell enclosing the distal tibia and fibula, a fabric attachment to footwear and a nylon cord connecting the shell to the footwear. Laboratory‐based stress tests using 3D computed tomography have shown that the device successfully provides ankle stability by limiting both excessive plantarflexion motion and the combined inversion–plantarflexion motion responsible for ankle instability, without limiting normal plantarflexion or dorsiflexion (Figure 2) [18]. A 3D scan was performed to determine the participants' measurements for manufacture of their brace(s). This involved taking a scan of the participant's feet and ankles using an iPad with a 3D Structure Sensor (XRPro LLC). Scans were then sent to the manufacturer (CADCAM Orthotics) and converted into a modelling software (LaserCam Orthotics) that created STL files of the braces. These files were then sent to a 3D printer (Formlabs Fuse 1+ 30W) and printed in Nylon 11 Powder using selective laser sintering and multi jet fusion (MFJ) methods (Figure 3). Participants with unilateral footdrop were fitted for one device, whereas participants with bilateral footdrop were fitted for two devices. Participants were fitted with their 3D‐printed custom ankle brace (approximately 3–4 weeks later). Fabric patches were added to the upper side of the shoes to allow attachment of the brace.

Participants were provided with a 4‐week wear‐in period to get used to the brace prior to testing. This 4‐week time frame was chosen to allow a sufficient period of familiarisation with the brace, as same‐day testing of ankle braces and ankle foot orthoses in people with CMT may not be enough to change usual locomotion patterns [19, 20]. Participants were also provided with a diary during the wear‐in period to record: (1) the number of hours they wore the device each day, (2) any adverse events (e.g., pain and skin irritation) and (3) any trips/falls. Participants were given the option of attending a podiatry visit if serious adverse events occurred. The 4‐week wear‐in period also allowed a longer time period for participants to consider acceptability of the brace during usual day‐to‐day activity.

### 2.5 Outcomes

To assess the effect of the 3D‐printed ankle brace on lower limb function (Aim 1), the following primary outcomes, which were chosen due to their feasibility in routine clinical practice, were assessed in both experimental conditions:

- The 10‐m walk test (10MWT) was used as a measure of walking speed [21, 22]. A 10‐m straight line was marked on a level surface with additional markers at 2 and 8 m. The participant was asked to walk along the full 10‐m length at a self‐selected speed, using whatever aids were needed. The clinician started a stopwatch when any part of the participants leading foot crossed the first mark (2 m) and stopped the stopwatch when any part of the leading foot crossed the end mark (8 m). Walking velocity (m/s) was calculated based on the time (seconds) taken to walk 6 m. The average of three trials was used for analysis.
- To assess balance, two balance subtest items from the Bruininks–Oseretsky Test of Motor Proficiency Second Edition (BOT‐2) [23] were used: standing with feet apart on a line and standing on one leg. Participants were instructed to perform each test with eyes open and then with eyes closed. The time that the participant maintained the position for each test was recorded, with a maximum cut‐off of 20 s for single leg and 30 s for double leg (i.e., if participants were able to maintain the single‐leg position for ≥20 s, then ‘20 s’ was recorded).
- The timed‐up‐and‐go test was also used as a performance‐based measure of lower limb function, mobility and falls risk [24, 25]. The participant was asked to start seated with their back against a standard height chair, without armrests. At the start of the timer and the clinician's ‘Go!’ command, the participant stood up from the chair and walked at a normal comfortable pace for 3 m to a line on the floor, where they turned around and returned to a seated position on the chair. The clinician stopped the timer when the participant's buttocks touched the chair. The average of two trials was used for analysis.

Secondary Aim 1 outcomes (also assessed during both experimental conditions) included the following patient reported outcomes:

- The 100‐mm visual analogue scale (VAS) was used to capture average foot pain in the past month (anchored with ‘no pain’ on the left and ‘extreme pain’ on the right).
- The 10‐item Manchester Foot Pain and Disability Index [26] was used to measure foot‐related pain and disability over the past month. Scores across all items were summated to give a total score out of 38, with higher scores indicating greater foot‐related pain and disability.
- The 14‐item Modified Falls Efficacy Scale (mFES) [27] was used as a measure of balance and mobility dysfunction. Scores were summated to give a total score out of 140 with higher scores reflecting more confidence/less fear of falling.

To assess the acceptability of the 3D‐printed custom ankle brace (Aim 2), the following outcomes were measured after the 4‐week wear‐in period: a 100‐mm VAS for overall comfort of the brace (anchored on the left with ‘not comfortable at all’ and on the right with ‘extremely comfortable’), a 100‐mm VAS for overall aesthetics of the brace (anchored on the left with ‘not happy with the look of the brace at all’ and on the right with ‘extremely happy with the look of the brace’) and a 100‐mm VAS for overall satisfaction of the brace (anchored on the left with ‘not satisfied at all’ and on the right with ‘extremely satisfied’). Data collected in the daily diaries related to adverse events and trips/falls were included under this aim.

### 2.6 Sample size

Using the PASS 15 software and a repeated measures analysis, an estimated 10 participants would allow the detection of a pre–post difference equivalent to 0.2 m/s (walking speed), assuming an SD of 0.2 and an autocorrelation among the repeated measurements of 0.5. The estimated difference and SD were obtained from previous work [28].

### 2.7 Data analysis

All participant demographic and medical data were described as mean (SD) for continuous data or frequency (%) for categorical data. To assess the difference in continuous functional variables between the two experimental conditions (Aim 1), a linear mixed models analysis was undertaken. The functional variables were included as the dependent variable with experimental condition (usual shoes only, usual shoes with brace) as the independent variable. The average hours worn per day (obtained from daily wear diaries) were included as a covariate, and the participant identification number was included as a random effect. For the single‐leg balance tests that were assessed on both right and left limbs, an additional participant‐specific random effect for limb side was included to account for the high level of dependence between right and left limbs within the individual. This analysis provided estimated differences between the two experimental conditions for all functional variables while adjusting for average wear time and accounting for the repeated measurements within each person. A similar approach was used for analysis of secondary Aim 1 outcomes. For each variable, Cohen's *d* were also calculated as a measure of effect size, with small, medium and large effect sizes corresponding to values of 0.2, 0.5 and 0.8 respectively [29]. In addition, minimal clinically important differences (MCID) for each variable were computed (MCID = 0.5 × footwear only SD) [30]. All data analysis was undertaken in SPSS at a 5% level of significance.

Variables relating to patient acceptability (Aim 2) were summarised descriptively as mean (SD) for continuous data or frequency (%) for categorical data. Subjective data from daily diaries related to acceptance and tolerability were described using content analysis.

## 3 RESULTS

### 3.1 Participant characteristics

Ten participants were included, with an age range of 18–64 years (Table 1). Mean duration of CMT was 22.8 years. Most participants had bilateral footdrop (90%) and protective sensory loss (80%). Mean number of falls per month was 12.1. Eight participants reported previous foot surgery, including toe arthroplasty (*n* = 3), forefoot reconstruction (*n* = 1), tendon lengthening (*n* = 1) and rearfoot arthrodesis (*n* = 3).

**TABLE 1:** Participant characteristics.

Age, years	
Mean (SD)	48.2 (14.0)
Range	18–64
Gender, *n* (%)	
Male	4 (40%)
Female	6 (60%)
Ethnicity, *n* (%)	
Māori	2 (20%)
European	8 (80%)
BMI, mean (SD)	26.7 (5.4)
CMT disease duration, years	
Mean (SD)	22.8 (18.2)
Range	1–55
CMT classification	
CMT 1	4 (40%)
CMT 5	1 (10%)
Other	1 (10%)
Unknown	4 (40%)
Foot drop, *n* (%)	
Bilateral	9 (90%)
Unilateral	1 (10%)
Walking aid use, *n* (%)	4 (40%)
Foot orthotic use, *n* (%)	4 (40%)
Previous foot surgery, *n* (%)	8 (80%)
FPI, mean (SD)	
Right	−5.4 (3.0)
Left	−2.7 (6.1)
Loss of protective sensation	
Right	8 (80%)
Left	8 (80%)
Number of falls in past month, mean (SD)	12.1 (27.2)
Any fall in past month, *n* (%)	5 (50%)

Abbreviations: BMI, body mass index; CMT, Charcot‐Marie‐Tooth disease; FPI, foot posture index.

### 3.2 Aim 1: Effectiveness of the 3D‐printed custom ankle brace

Differences in lower limb function outcomes between the two experimental conditions are shown in Table 2. A statistically significant improvement in balance with eyes open was observed when using the brace (*p* = 0.026) with a medium effect size and an MCID of 1.63 s. All other lower limb function outcomes did not show a significant difference (all *p* > 0.05).

**TABLE 2:** Effect of the 3D‐printed custom ankle brace on lower limb function (primary Aim 1 outcomes).

	Mean estimates^a^, seconds			95% CI		*p*	Cohen's *d*	MCID
	Usual footwear	Usual footwear with brace	Diff.	Lower	Upper			
10‐m walk test, m/s	8.6	8.1	−0.4	−1.7	0.8	0.42	0.33	0.76 m/s
Balance, both legs, eyes open and seconds	27.9	28.1	0.2	−5.8	6.3	0.94	0.02	4.47 s
Balance both legs, eyes closed and seconds	22.7	26.7	4.0	−4.1	12.1	0.28	0.37	5.40 s
Balance, single leg^b^, eyes open, seconds	7.5	9.3	1.7	0.2	3.2	**0.026**	0.55	1.63 s
Balance single leg^b^, eyes closed and seconds	1.7	2.0	0.3	−0.3	1.0	0.30	0.21	0.70 s
Time up and go, mean (SD) and seconds	8.6	8.0	−0.5	−1.3	0.3	0.16	0.61	0.49 s

*Note:* Bolded values indicate statistical significance at p 〈 0.05.

Abbreviation: MCID, minimal clinically important difference.

^a^ Mean estimates are adjusted for hours worn over the 4‐week wear‐in period.

^b^ Adjusted for repeated measures on right and left limbs.

Comparison of the two experimental conditions for patient reported outcomes revealed a significant reduction in foot pain with use of the brace (*p* = 0.45) (Table 3). The effect size for change in foot pain was large and the MCID was 10.98 mm. All other patient reported outcomes showed no significant difference (all *p* > 0.05).

**TABLE 3:** Effect of the 3D‐printed custom ankle brace on patient reported outcomes (secondary Aim 1 outcomes).

	Mean estimates^a^			95% CI		*p*	Cohen's *d*	MCID
	Usual footwear	Usual footwear with brace	Diff.	Lower	Upper			
Foot pain VAS, mm	45.8	27.8	−18.0	−34.4	−0.5	**0.045**	0.82	10.98 mm
MFPDI	20.4	18.7	−1.8	−5.3	1.7	0.27	0.36	2.35
MFES	118.1	118.7	0.6	−12.7	13.8	0.92	0.03	8.84
Number of falls in past month^b^	3.3	0.0	−3.3	−11.7	4.9	0.37	0.32	5.23 falls

*Note:* Bolded values indicate statistical significance at p 〈 0.05.

Abbreviations: MCID, minimal clinically important difference; MFES, Modified Falls Efficacy Scale; MFPDI, Manchester Foot Pain and Disability Index; VAS, Visual Analogue Scale.

^a^ Mean estimates are adjusted for hours worn over the 4‐week wear‐in period.

^b^ Based on data from 8 participants who had completed diary data.

### 3.3 Aim 2: Acceptability of the 3D‐printed custom ankle brace for people with CMT

Comfort and satisfaction levels between participants varied considerably (Table 4). Overall mean (range) comfort of the ankle brace on the 100‐mm VAS was 62.7 (32–86) and overall satisfaction was 73.9 (28–95). Subjective feedback recorded in the daily diaries also showed that one participant found the brace too firm about the ankle malleoli due to the absence of soft tissue mass around this area. Two participants also found it challenging to handle the braces due to a loss of dexterity in their hands due to CMT‐related neuropathy affecting the upper limb. This made it difficult to don and doff the brace. One participant also noted that given the amount of time they wear the brace, having a shoe mounted fixing and associated connecting cord in colours that could match their shoe would likely increase acceptance. There were no participants in this study who required podiatry appointments during the wear‐in period.

**TABLE 4:** Patient acceptability (Aim 2 outcomes).

	Mean (SD)
Overall comfort, VAS, mm, mean (SD), range	62.7 (17.9), 32–86
Look of the brace, VAS, mm, mean (SD), range	61.6 (21.1), 19–82
Overall satisfaction, VAS, mm, mean (SD), range	73.9 (21.2), 28–95
Hours worn over 4 weeks, mean (SD)	49.5 (36.1)
Hours worn weekly, mean (SD), hours	14.4 (10.1)
Falls during study period, *n* (%)	0 (0%)

Abbreviation: VAS, visual analogue scale.

## 4 DISCUSSION

This is the first study investigating the role of a 3D‐printed custom ankle brace in people with CMT. The results from this pilot study suggest that the brace may improve balance and reduce foot pain in people with CMT following a 4‐week wear‐in period. Overall comfort and satisfaction with the brace varied across participants.

Single‐leg balance time with eyes open was significantly increased when wearing the brace. The single‐leg stance test is used to test balance and postural control as well as quantify falls risk [31]. The improved balance observed in the current study may have been through the improved proprioceptive feedback provided by the brace. Most participants demonstrated distal sensory loss, which is consistent with the progressive neuropathic nature of CMT in which sensation reduces from distal to proximal [2]. Previous research has also shown that the use of an ankle brace influences ankle joint behaviour by allowing wearers to adapt their foot position more efficiently in anticipation of the nature of the landing surface [32]. The current results are also consistent with stroke research, in which mediolateral ankle bracing has been shown to improve static and dynamic balance in stroke patients [33]. This research, however, did not find a significant difference in single‐leg balance with eyes closed, which may suggest that people with CMT rely on visual cues to maintain balance and postural control [34].

This study also found a significant reduction in foot pain when wearing the brace. This finding may be explained by the predominance of the pes cavus (supinated) foot type in the participants and the mechanism of action of the brace. Cavus foot deformity in people with CMT is associated with widespread foot pain and increased pressure under the forefoot and midfoot [35]. By reducing footdrop, the brace changes the foot‐ground contact pattern during gait and may provide therapeutic benefit to people experiencing pressure‐induced foot pain. The footdrop function of the brace likely has an ability of ‘slowing down’ the eccentric or braking phase of the foot extensors at contact and has been shown to reduce metabolic load in people with CMT [20]. The lateral stabilising effect of the brace may also support the muscles and ligaments providing lateral stability to the foot.

There was a wide variation in reported comfort of the brace by participants that may have impacted time spent wearing the brace throughout the wear‐in period. Issues with braces in people with CMT are common [14]. The 3D‐printed brace used in the current study (EXO‐L UP) is an adaptation of its related EXO‐L brace whose main indication is the prevention of lateral ankle sprains in athletes. People with CMT using the brace are more likely to wear the brace for longer periods of time than a typical ankle brace user. The moderate levels of comfort and satisfaction reported by participants in the current study are similar to those reported in studies assessing custom‐made polypropylene and silicone ankle foot orthoses in people with CMT [9]. These findings suggest that the design of the brace could potentially be refined and customised further to improve acceptance and tolerance in people with CMT, including considering hand dexterity and soft tissue wasting around the ankles. Due to the progressive changes in the lower limb with CMT, having a greater ability to customise the brace may also increase acceptance and use.

This study has some strengths and limitations. Although the sample size was small, it was sufficiently powered to detect significant differences in primary outcomes. The sample size is also similar to other within‐subject comparison studies of people with CMT assessing ankle foot orthoses and braces which range from 3 to 12 participants [9, 20, 36, 37, 38, 39, 40]. Nevertheless, it should be acknowledged that the current study was a pilot study and is an initial step in exploring 3D‐printed ankle braces for people with CMT. The results from this study may be used to inform feasibility and identify modifications needed in the design of larger, ensuring hypothesis testing studies. Participants in this study were also representative of the typical CMT population in New Zealand in terms of clinical characteristics. A potential limitation of this study was that participants' footwear was not controlled for, which may have influenced the function of the brace. However, allowing participants to wear their own footwear improves adherence and is more reflective of the footwear they wear outside of research settings. This is consistent with previous studies examining the use of ankle braces by people with CMT, in which participants prefer to wear their own footwear [14]. Finally, participants in the current study wore the brace for an average for 14 h per week during the wear‐in period, and it may be that longer use of the brace would have a larger impact on patient‐reported and functional outcomes. The 4‐week wear‐in period used in this study may not have been long enough for participants to wear‐in or get used to the brace. Future controlled trials assessing efficacy of ankle braces in CMT over longer time periods are required. In addition, further research examining the impact of 3D‐printed ankle braces on spatiotemporal variables, frontal and sagittal plane kinematics, plantar pressure and other functional tests, such as stair climbing, may provide further insight into the dynamic function of the brace. Further research comparing 3D‐printed ankle braces with both prefabricated and customised non‐3D‐printed ankle braces would also be of interest to determine the impact of 3D‐printing customisation on both function and acceptability in this population.

In conclusion, this pilot study suggests that a 3D‐printed ankle brace may improve balance and foot pain in people with CMT. However, larger scale studies are warranted to further explore the impact of the brace on function and balance outcomes along with the long‐term effects of the brace in people with CMT. Future work should also consider customisation of the brace and its impact on patient acceptability.

## AUTHOR CONTRIBUTIONS

**Adam Philps**: Formal analysis; investigation; methodology; resources; visualisation; writing—original draft preparation; writing—review & editing. **Mike Frecklington**: Methodology; project administration; supervision; writing—review & editing. **Sarah Stewart**: Formal analysis; methodology; project administration; supervision; writing—review & editing.

## CONFLICT OF INTEREST STATEMENT

The authors declare that there are no conflicts of interest.

## ETHICS STATEMENT

Ethical approval was obtained from AUT Ethics Committee (AUTEC 22/91). All participants were required to provide written informed consent prior to participation.

## Data Availability

Data are available on request due to privacy/ethical restrictions. The data that support the findings of this study are available on request from the corresponding author. The data are not publicly available due to privacy or ethical restrictions.
